# Human Adipose-Derived Stem Cells Promote Seawater-Immersed Wound Healing by Activating Skin Stem Cells via the EGFR/MEK/ERK Pathway

**DOI:** 10.1155/2019/7135974

**Published:** 2019-12-31

**Authors:** Jiachao Xiong, Boyao Ji, Liujun Wang, Yazhou Yan, Zhixiao Liu, Shuo Fang, Minjuan Wu, Yue Wang, Jianxing Song

**Affiliations:** ^1^Department of Plastic Surgery, Changhai Hospital, Naval Military Medical University, Shanghai 200433, China; ^2^Department of Histology and Embryology, Naval Military Medical University, Shanghai 200433, China; ^3^Shanghai Key Laboratory of Cell Engineering, Shanghai, China; ^4^Department of Neurosurgery, Changhai Hospital, Naval Military Medical University, Shanghai 200433, China

## Abstract

Seawater (SW) immersion can increase the damage of skin wounds and produce refractory wounds. However, few studies have been conducted to investigate the mechanisms of SW immersion on skin wounds. In our current study, we investigated the effect of human adipose-derived stem cells (hADSCs) on the repair of SW-treated full-thickness skin wounds and the underlying mechanisms. The results showed that SW immersion could reduce the expression of EGF and suppress the activation of the MEK/ERK signaling pathway. At the same time, the proliferation and migration of skin stem cells were inhibited by SW immersion, resulting in delayed wound healing. However, hADSCs significantly accelerated the healing of SW-immersed skin wounds by promoting cell proliferation and migration through the aforementioned mechanisms. Our results indicate a role for hADSCs in the repair of seawater-immersed skin wounds and suggest a potential novel treatment strategy for seawater-immersed wound healing.

## 1. Introduction

Chronic wounds are wounds that do not reach anatomical and functional integrity within 30 days after injury [1]. Diabetes, obesity, persistent infection, and the use of corticosteroids can make skin wounds difficult to heal and can lead to chronic skin wounds, which may eventually lead to serious consequences such as infection, amputation, and even death [2, 3]. Seawater (SW) immersion is also a common cause of chronic wounds in people living in coastal areas and involved in ocean navigation. SW, a complex hypertonic alkaline solution whose chemical composition is mainly NaCl, also includes different proportions of KCl, CaCl_2_, MgCl_2_, MgSO_4_, and the like. Global SW has an average salinity of 34.7 and a pH of 8-8.4, which is a pronounced hyperosmotic alkaline state. In addition, SW contains a large number of microorganisms, especially Gram-negative bacteria [4]. The above characteristics mean that when skin wounds are soaked in SW for a long time, they become prone to tissue necrosis and infection, prolonging the healing time of the skin wounds and causing chronic wounds. Regrettably, there have been rare reports on the effects of SW on the wounds of full-thickness skin and the mechanism of its occurrence.

Stem cell therapy has become a new direction for the treatment of chronic wounds. Human adipose-derived stem cells (ADSCs) are multidirectional differentiation potential stem cells extracted from adipose tissue. ADSCs can migrate to a damaged site and differentiate into skin appendages to repair damaged skin through their multidirectional differentiation potential [5–8]. At the same time, ADSCs can secrete various growth factors to inhibit the inflammatory response, accelerate wound angiogenesis, and promote wound healing [9]. ADSCs can also be used as seed cells that work with innovative repair materials; ADSCs can grow in 3D culture on injectable hydrogel scaffolds, which was reported to increase the retention rate of ADSCs, promote wound angiogenesis, and accelerate the healing of chronic wounds [10, 11]. However, there is no report on the application of ADSCs in SW immersion wound repair.

In this study, we established a wound model of SW immersion and compared it with normal wound healing; comparing the two conditions, we confirmed that SW immersion could significantly delay wound healing. Skin stem cells are one of the important cell types in wound healing. Skin stem cells can gradually move up from the basal layer and differentiate into epidermal progeny cells to promote wound healing [12]. We hypothesized that ADSCs could promote the repair of SW-soaked wounds by differentiating into skin stem cells and promoting the proliferation and migration of autologous skin stem cells. Previous studies have shown that EGF is the most important growth factor for skin reepithelialization. Furthermore, the expression of EGF can activate the MEK/ERK signaling pathway and promote cell proliferation and migration. Therefore, we believe that ADSCs can promote the proliferation and migration of skin stem cells and accelerate the process of wound closure by regulating the expression of EGFR and the activation of the MEK/ERK pathway, which illustrate new treatment strategies for wound healing.

## 2. Materials and Method

### 2.1. Cell Isolation and Culture

Human subcutaneous adipose tissue samples were obtained from the abdominal liposuction of 10 healthy women in the Changhai Hospital affiliated with the Second Military Medicine University. All samples were obtained and used with informed consent of the patient. Next, the adipose tissue samples were washed with low-glucose DMEM (HyClone, Utah, USA) to remove excessive lipid droplets. Next, the samples were digested with 0.075% type II collagenase (Gibco, Grand Island, USA) for 40 minutes with gentle agitation at 37°C. The samples were filtered with 70 *μ*m mesh filters, mixed with low-glucose DMEM supplemented with 10% FBS (Gibco, Grand Island, USA) and 1% P/S (Gibco, Grand Island, USA), and then centrifuged at 300×g for 30 minutes. The hADSC fraction was washed with PBS (HyClone, State of Utah, USA) and centrifuged again at 300×g for 10 minutes, and then the supernatant was discarded. The cell pellet was resuspended in low-glucose DMEM supplemented with 10% FBS and cultured in a humidified incubator with 5% CO_2_. After the hADSCs were attached, the medium was changed every 2-3 days [13].

HaCaT cells are representative human epidermal cell lines. We obtained HaCaT cells from the American Type Culture Collection (Manassas, VA, USA). HaCaT cells were cultured in high-glucose DMEM (HyClone, Utah, USA) supplemented with 10% FBS, and the medium was changed every 2-3 days.

### 2.2. Characterization of hADSCs

#### 2.2.1. Flow Cytometric Characterization

After 3 passages, the hADSCs were collected and counted. Approximately 1 × 10^6^ cells were washed and labeled with fluorophore-conjugated antibodies (BD Biosciences, USA) (CD105-FITC, CD90-PE-CyTM7, CD29-APC, CD49d-PE, CD45-PE-CyTM7, and CD34-PE) and incubated for 20 minutes at room temperature [14]. The cells were stained with an isotype control IgG as a control. The cells were then washed with PBS and analyzed by flow cytometry (Amnis ImageStreamX Mark II, Seattle, USA).

#### 2.2.2. Multidirectional Differentiation Characterization

The hADSCs were cultured with osteogenic (Cyagen, Guangzhou, China) or adipogenic (Cyagen, Guangzhou, China) differentiation medium for 4 weeks. Then, the cells were fixed with 4% neutral formaldehyde for 30 min and washed twice with PBS. Then, the cells induced to osteogenic differentiation were stained with alizarin red S (Cyagen, Guangzhou, China) for 5 min, and the cells induced to adipogenic differentiation were stained with oil red O (Cyagen, Guangzhou, China) for 30 min at room temperature. The stained cells were washed with PBS, and images were captured using a light microscope (Olympus, Japan) to observe and evaluate the status of adipogenic differentiation or osteogenic differentiation.

### 2.3. SW Solution Preparation

The experimental SW solution was configured according to the standard of the Third Institute of Oceanography of the State Ocean Bureau. The SW components were as follows: NaCl 26.52 g/l, MgCl_2_ 22.45 g/l, MgSO_4_ 3.31 g/l, CaCl_2_ 1.14 g/l, KCl 0.73 g/l, NaHCO_3_ 0.202 g/l, and NaBr 0.083 g/l. The SW formulations were as follows: osmotic pressure (1250.00 ± 11.52) mOsm/l, pH 8.20, Na^+^ (630.00 ± 5.33) mmol/l, K^+^ (10.88 ± 0.68) mmol/l, and Cl^−^ (658.80 ± 5.25) mmol/l, and the average salinity of the seawater was 35%. Experimental SW was filtered through a 0.22 *μ*m filter before use.

### 2.4. *In Vivo* Studies

A total of 48 6-week-old Balb/c male nude mice were randomly divided into a control group, an SW group, an SW+DMEM group, and an SW+hADSC group. Among them, the control group had no intervention, while the SW group only had seawater immersion without other intervention. The control group was compared with the SW group to prove that seawater immersion could delay wound healing and form a chronic wound. Then, the SW+hADSC group was compared with the SW group to prove that hADSCs could significantly promote the healing of seawater-immersed wounds. Since injected hADSCs were suspended in DMEM, the SW+DMEM group was established to rule out the effect of DMEM on wound repair and to confirm that hADSCs are the key cells for repair. All the experimental protocols in this study were approved by the guidelines of the Health Sciences Animal Policy and Welfare Committee of Changhai Hospital affiliated with the Second Military Medical University.

**Figure 1:**
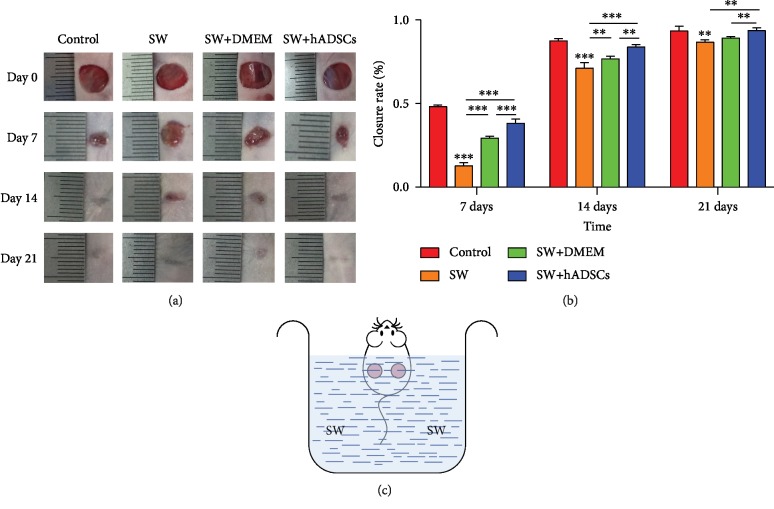
*In vivo* observation of wound healing after SW and hADSC treatments. The wound area was measured and calculated on days 7, 14, and 21 after injury with Image-Pro Plus 6.0 software (a). The SW-immersed wounds showed a slower rate of reepithelialization and closure during the healing period on the 7th and 14th days, while the hADSC injection significantly accelerated the healing of SW-immersed wounds (b). ^∗^*P* < 0.05, ^∗∗^*P* < 0.01, and ^∗∗∗^*P* < 0.001. Schematic diagram of the seawater immersion model (c).

First, a full-thickness skin defect model was established. Mice were anesthetized by subcutaneous injection of a mixture of Zoletil (0.1 ml/kg) and Rompun (0.05 ml/kg). A pair of full-thickness skin wounds was created by two 8 mm biopsy punches on the lateral back of the mice [15]. Subsequently, the wound surface of the mouse was completely immersed in a measuring cup filled with seawater for 60 minutes to establish a seawater-soaked wound model (schematic diagram shown in Figure 1(c)). For the control group, the mice did not receive any other intervention; for the SW group, the wounds of mice were immersed in SW without other intervention; and for the SW+DMEM and SW+hADSC groups, the wound surfaces were immersed in SW. Then, the residual seawater on the surface of the mouse was gently wiped off with a clean gauze. After the mice were stable (approximately 10 minutes), the SW-immersed wounds were injected with 100 *μ*l of low-glucose DMEM or 1 × 10^6^ hADSCs that were suspended in low-glucose DMEM at six injection sites at the subcutaneous base of the wound. Finally, Vaseline gauze was used to cover the wound surface of the mouse, and the gauze was pressure bandaged. All mice were fed in separate cages. On the third day after dressing, the subcutaneous base of the SW-immersed wounds received multipoint injections with 100 *μ*l of low-glucose DMEM or 1 × 10^6^ hADSCs that were suspended in low-glucose DMEM, after which no other therapeutic injection intervention was performed.

The wounds of each mouse were photographed digitally. The wound area was calculated from measurements taken on days 7, 14, and 21 after injury using Image-Pro Plus 6.0 software (Medical Cybernetics, USA). The wound healing rate was calculated as follows:

(1) $$\begin{array}{c} \text{The healing rate}=\frac{\left(\text{Area of initial wound}–\text{Area of residual wound}\right)}{\text{Area of initial wound}}\times 100\%. \end{array}$$

### 2.5. Immunohistochemistry and Immunofluorescence Studies

The wound skin tissues on days 14 and 21 were excised, and histological staining analysis was performed. The excised skin samples were fixed in 10% formalin and dehydrated by incubation in an ethanol gradient. The samples were embedded in paraffin, and 5 *μ*m thick sections were cut. Tissue sections were subjected to HE staining and Masson's trichrome staining for histological observation and assessment of collagen maturation status, respectively.

For *in vivo* studies, hADSCs were stained with red fluorescence using CM-Dil dye (Invitrogen, Thermo Scientific, USA) before skin wound injection treatment. The SW+hADSC group's wound skin tissues were excised on day 14 and were immediately placed on the tissue supporter. Then, the tissues were embedded in a tissue-embedding agent and frozen to prepare 10 *μ*m thick frozen section.

Immunohistochemistry and immunofluorescence assays were performed as previously reported [14]. The main primary antibodies used in this experiment were CK19 (Proteintech, Wuhan, China) and Ki67 (Abcam, Cambridge, MA, UK). As a measure of skin wound repair, CK19 staining was performed to assess the proliferation of skin stem cells; Ki67 staining was performed to assess proliferating cell levels. The images of stained sections were collected by photographing using an optical microscope (Olympus, Japan). Five areas were randomly selected for each of the sections, and cells were counted using Image-Pro Plus 6.

For immunofluorescence, first, the tissue sections were incubated with primary antibodies at 4°C overnight. Next, the sections were incubated with secondary antibodies at room temperature for an hour. The secondary antibodies were Alexa Fluor™ 594 goat anti-rabbit IgG (H+L) cross-adsorbed secondary antibody (Invitrogen, Thermo Scientific, USA) and FITC-labeled goat anti-rabbit IgG (H+L) cross-adsorbed secondary antibody (Beyotime Biotechnology Company, Jiangsu, China). Then, the sections were stained with an antifade mounting agent (ProLong, Thermo Scientific, USA) containing DAPI. Finally, the sections were covered with glycerin, and a coverslip was added. The above operations were carried out in the absence of light when possible. The fluorescent staining sections were photographed using a fluorescence microscope, and the counting method was performed as described above [16].

### 2.6. Cell Treatment

To explore the effects of SW treatment on HaCaT cells, the cells were cultured in high-glucose DMEM supplemented with 10% FBS. The cells were divided into four groups and mixed into seawater with a 0%, 1.75%, 3.5%, and 7% salt concentration.

**Figure 2:**
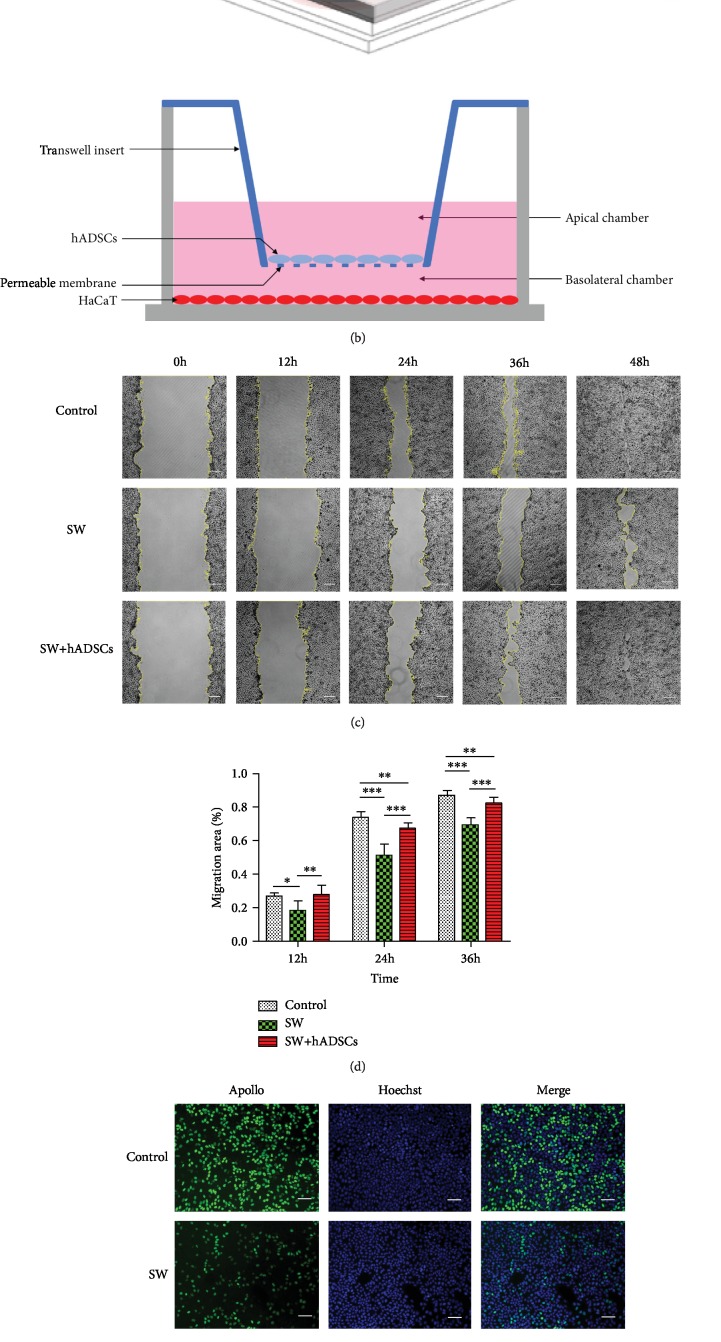
Effect of hADSCs on the proliferation and migration of SW-treated cells *in vitro*. CCK8 assay results showed that the proliferation of HaCaT cells was significantly inhibited following exposure to 3.5% salt concentration (a). Transwell coculture system schematic (b). The cell scratch test (c) showed that the SW group showed a significant closure delay at the 12th hour compared with the control group, and the hADSC coculture reduced the effect of SW on cell migration ability and significantly promoted cell migration (d). Proliferating HaCaT cells were stained with green fluorescence, and all nuclei were stained with blue fluorescence (e). The results of the EdU assay showed that the cell proliferation rate in the SW group was significantly inhibited compared with that in the control group and the SW+hADSC group (f). Scale bars indicate 100 *μ*m. ^∗^*P* < 0.05, ^∗∗^*P* < 0.01, and ^∗∗∗^*P* < 0.001.

To investigate the effects of hADSCs on SW-treated cells, HaCaT cells were cocultured with hADSCs in six-well plates and using a transwell system (schematic diagram shown in Figure 2(b)). The hADSCs (1 × 10^5^ cells/well) were seeded onto a 0.4 *μ*m polycarbonate membrane in a transwell (Corning Costar, Cambridge, MA); HaCaT cells (1 × 10^4^ cells/well) were plated in the lower chambers, and the cells were cultured together for 2 days.

### 2.7. Cell Proliferation Assay

CCK8 assays (Beyotime Biotechnology Company, Jiangsu, China) were used to detect the effect of SW on cell proliferation. HaCaT cells were seeded at 2,000 cells/well in 96-well plates. After 12 hours of allowing the cells to attach, the cells were cultured with different concentrations of salt (0, 1.75%, 3.5%, and 7%). Cell growth was analyzed 24 h, 48 h, and 72 h after treatment. A 10 *μ*l CCK8 test solution was added to each well, and the cells were incubated in a humidified incubator with 5% CO_2_ at 37°C for 2 h at each time point. The OD was measured at 450 nm with a microplate reader (Tecan, Thermo Scientific, USA). The data shown are representative of four independent experiments. Based on these data, we determined a suitable salt concentration for the follow-up experiment.

An EdU kit (Ribo, Guangzhou, China) was used to detect the effect of hADSCs on the proliferation of SW-treated cells [17]. The HaCaT cells were seeded at 1 × 10^5^ cells/well in 24-well plates and cultured normally until their confluency was 70-80%. Then, the cells were divided into three groups: a normal group, an SW group, and an SW+hADSC group. The normal group of cells were cultured in high-glucose DMEM. The SW group and SW+hADSC group of cells were cultured in high-glucose DMEM mixed with 3.5% salt. Moreover, the SW+hADSC group of cells were cocultured with hADSCs using 0.4 *μ*m transwell polycarbonate membranes [18]. After the cells were cultured for 48 h, the EdU experiment was performed. First, EdU reagent was added to the cell culture medium at a dilution ratio of 1000 : 1, and the cells were incubated for 2 h. Next, the medium was discarded, and the cells were washed with PBS. Third, 500 *μ*l of 4% paraformaldehyde was added to each well and was followed by incubation for 30 min, and then 500 *μ*l of 2 mg/ml glycine solution was added to neutralize excess formaldehyde. Next, each well was supplemented with 0.5% TritonX-100 or 10 min to permeabilize the cells, and then they were washed with PBS for 5 min. For cytofluorescence staining, a 1x Apollo reaction solution was added to the culture dish, and the cells were incubated for 30 min at room temperature in the dark. Next, each well was supplemented with 0.5% TritonX-100 or 10 min to permeabilize the cells. Finally, a 1x Hoechst 33342 reaction solution was added to the culture dish, and cells were incubated at room temperature for 30 min in the dark and then observed with a Zeiss fluorescence microscope (HLA100, Shanghai, China).

### 2.8. Cell Migration Assay

Scratch tests were used to evaluate the effect of SW on cell migration and the effect of ADSCs on the migration ability of SW-treated cells [19]. The HaCaT were still divided into three groups: normal group, SW group, and SW+hADSC group. The cells were inoculated in 6-well plates at 1 × 10^6^ cells/well, and they were cultured normally until the cells were 80%-90% confluent. Then, the confluent cell monolayer was scratched with a sterile 10 *μ*l pipette tip, and the cells were washed with PBS. Fresh medium was then added to the cells, and the culture method was the same as in the above EdU experiment. Cells were observed, and images were recorded with a Zeiss inverted phase contrast microscope (Observer. D1, Shanghai, China) at 0, 12, 24, and 36 hours after scraping the cells. The migration area was measured by using ImageJ software (Bethesda, MD, USA). The following formula was used to evaluate cell migration ability:

(2) $$\begin{array}{c} \text{Migration area }\left(\%\right)=\frac{\text{A}0-\text{Ax}}{\text{A}0}\times 100\%, \end{array}$$

where A0 represents the initial scratch area (*t* = 0 h), and Ax represents the residual scratch area at the time of measurement (*t* = *n* h).

### 2.9. Western Blotting

Skin wound tissues from each group on day 14 and day 21 were isolated, and samples were washed with PBS. Then, the tissue lysate was added to completely lyse the tissue, and the lysate was centrifuged at 1,200 g for 10 minutes to collect the supernatant solution containing the protein. Supernatants containing equal amounts of protein were added to a 10% SDS-PAGE gel, were electrophoresed, and were transferred to a PDVF membrane. The membrane was then blocked in 5% BSA for 2 h at room temperature and incubated overnight at 4°C in the primary antibody solution. The primary antibodies recognized GAPDH (Cell Signaling Technology, MA, USA) and EGFR (Cell Signaling Technology, MA, USA). Finally, the membrane was washed 3-4 times with TBS-T and incubated with a goat anti-rabbit HRP-conjugated secondary antibody (Cell Signaling Technology, MA, USA) for 2 h at room temperature. The intensity of protein expression was observed on an Alpha Imager scanner using chemiluminescence (Tecan, Thermo Scientific, USA), and expression analysis was performed using ImageJ software.

### 2.10. Quantitative Real-Time PCR

Extracting RNA from skin wound tissue. Total RNA was isolated by using Trizol® reagent (Life Technologies, USA) according to the manufacturer's instructions. One microgram of total RNA was reverse-transcribed into cDNA using the ReverTra Ace® qPCR RT Master Mix with gRNA Remover (TOYOBO, Osaka, Japan). Samples were assessed in quantitative real-time PCR reactions using the QuantStudio™ 7 Flex Real-Time PCR System (Life Technologies, USA) with SYBR® Green Real-time PCR Master Mix (TOYOBO, Osaka, Japan). PCR was performed with an initial step of denaturation at 95°C for 5 min, followed by 40 cycles of 95°C for 10 s, 60°C for 20 s, and 72°C for 20 s. Melt curves were established for the reactions, and normalized fold expression was calculated using the 2^-*ΔΔ*Ct^ method. The related primer sequences are listed in Table 1.

### 2.11. Statistical Analysis

All data are expressed as the mean ± SD, and one-way ANOVA was used for multiple group comparisons. All statistical analyses were performed using GraphPad Prism software (version 7.0, La Jolla, CA) and SPSS statistic software package version 15.0 (Chicago, IL, USA). *P* values < 0.05 were considered statistically significant.

## 3. Results

### 3.1. Characterization of hADSCs

**Figure 3:**
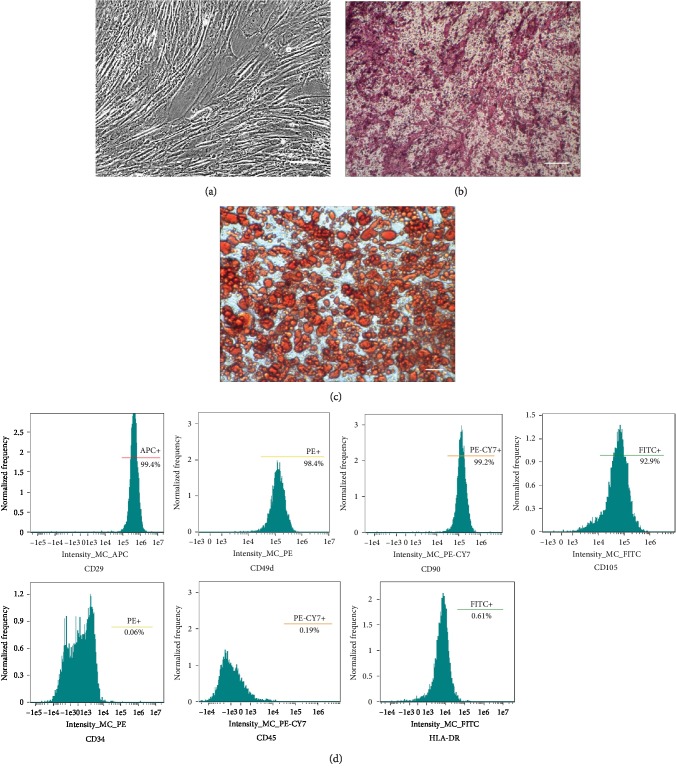
Characterization of hADSCs. Cell morphology of hADSCs (a), alizarin red S staining for osteoblasts (b), and oil red O staining for adipocytes (c). Scale bar indicates 100 *μ*m. Flow cytometry results showed that cells were strongly positive for CD29, CD105, CD90, and CD49d but negative for CD34 and CD45 (d).

According to previous research methods, hADSCs were extracted, and the osteogenic and adipogenic differentiation potentials were analyzed [14, 20]. Primary hADSCs began to adhere to wells, and cell morphology was typical after 3 to 7 days of culture, as shown in Figure 3(a). To confirm that hADSCs were successfully isolated from human adipose tissue, hADSCs were induced to differentiate into adipocytes and osteoblasts, and the adipogenic differentiation and osteogenic differentiation ability of cells was confirmed by alizarin red S staining of osteoblasts (Figure 3(b)) and oil red O staining of adipocytes (Figure 3(c)), respectively. Flow cytometry results (Figure 3(d)) showed that cells were strongly positive for the surface markers CD29, CD105, CD90, and CD49d, but they were negative for CD34 and CD45. The above characteristics were consistent with the previous research results, indicating that we obtained high-quality hADSCs.

### 3.2. The Effect of hADSCs on the Treatment of Seawater-Immersed Wounds

**Figure 4:**
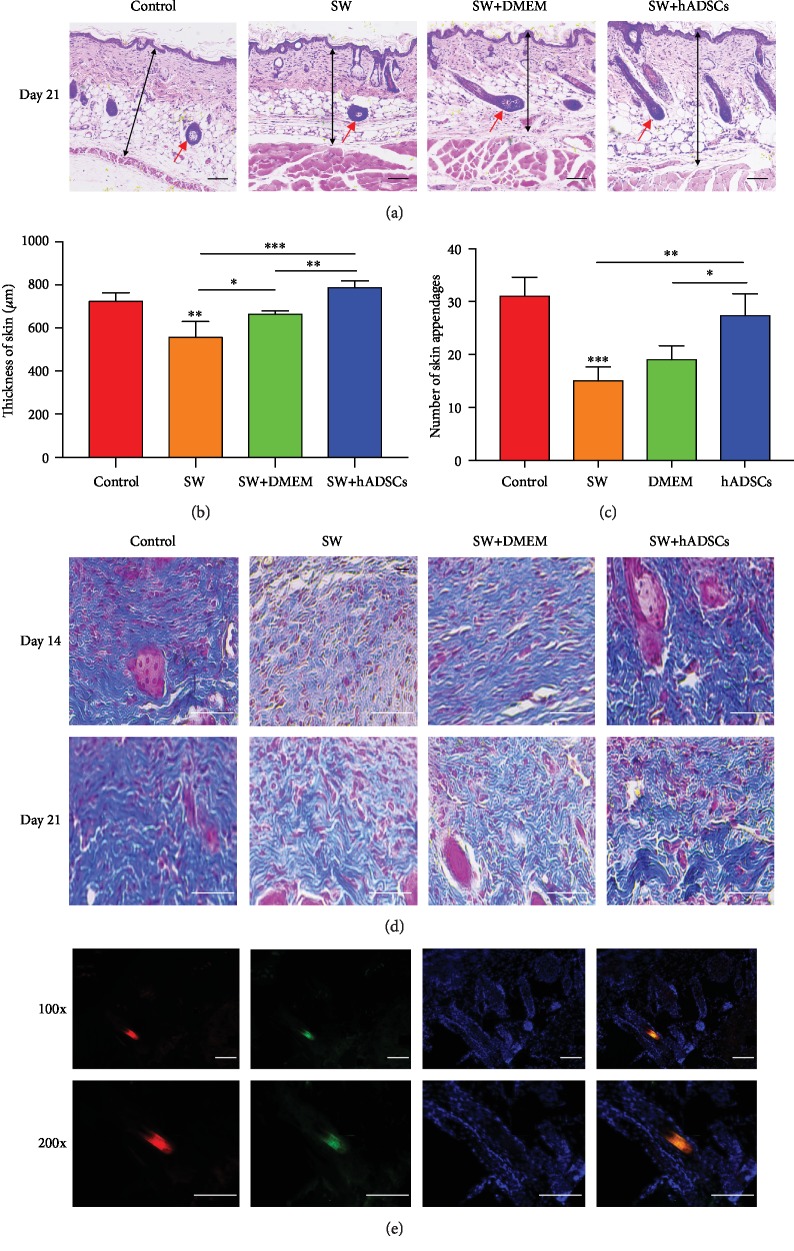
Microscopic evaluation of wound repair after SW and hADSC treatments. HE staining for wound repair, skin thickness, and number of subcutaneous appendages (a). The regeneration of skin thickness (b) and appendages (c) in the SW group and the SW+DMEM group was significantly decreased compared to that in the control group and the SW+hADSC group. Masson's trichrome staining of wounds in each group (d). Scale bars indicate 100 *μ*m. ^∗^*P* < 0.05, ^∗∗^*P* < 0.01, and ^∗∗∗^*P* < 0.001. The red arrow refers to a hair follicle. The frozen section immunofluorescence showed that hADSCs (CM-DIL, red) differentiated into skin stem cells (cytokeratin 19, CK19, green) to promote wound healing (e).

To assess the effect of hADSCs on the treatment of seawater-immersed wounds, we observed the healing process of mouse skin wounds (Figures 1(a) and 1(b)) and found the following: the wounds of mice immersed in SW showed a slower healing rate than those in the control group, and the SW+hADSC group showed a significantly higher wound healing rate (*P* < 0.001). Moreover, microscopic observation of wound sections (Figures 4(a) and 4(b)) showed that the skin in the SW group and the SW+DMEM group was significantly thinner than that in the control group and the SW+hADSC group (*P* < 0.01). At the same time, more collagen deposition and regeneration of skin appendages (Figures 4(c) and 4(d)), such as hair follicles and sweat glands, were observed in the control group and the SW+hADSC group compared with the SW group and the SW+DMEM group (*P* < 0.01).

First, we established a full-thickness skin defect model and immersed the wound completely in artificial seawater. During the healing period on days 7 and 14, the reepithelialization and closure of the seawater-immersed wounds were slower than what was observed in control wounds, demonstrating that seawater impedes wound healing [21]. Subsequently, hADSCs or an equal volume of DMEM were injected around the wounds, and it was observed that wounds with the hADSC injection had a better closure efficiency. Finally, tissue sections were used to further observe the morphological changes in wound repair [20]. It was also confirmed that seawater immersion hindered the morphological repair of wounds, and hADSCs reduced the impact of seawater on wounds and promoted their repair. Therefore, we believe that hADSCs can significantly promote the healing of seawater-immersed wounds.

### 3.3. Effect of hADSCs on the Proliferation and Migration of SW-Treated Cells *In Vitro*

The proliferation and migration of epidermal cells play a crucial role in wound healing [22], so we studied the effect of hADSCs on the proliferation and migration of SW-treated HaCaT cells. The CCK8 assay results of HaCaT cells (Figure 2(a)) showed that a low salt concentration (1.75%) could promote the proliferation of HaCaT cells to a certain extent. However, the proliferation of cells was significantly inhibited in the presence of 3.5% salt (*P* < 0.05). Therefore, the 3.5% salt concentration was used as the experimental concentration for subsequent studies.

EdU assays were used to evaluate the effect of SW and hADSCs on cell proliferation. The results (Figures 2(e) and 2(f)) showed that the cell proliferation rate in the SW group was significantly lower than the rate in the control group and the hADSC coculture group (*P* < 0.01).

Cell scratch tests were used to evaluate the effect of SW and hADSCs on cell migration. The results (Figures 2(c) and 2(d)) showed that the SW group showed a significant closure delay at the 12th hour compared with the control group (*P* < 0.05), and the hADSC coculture group reduced the effect of SW on cell migration ability and significantly promoted cell migration (*P* < 0.01). In addition, the control group and the hADSC coculture group of HaCaT cells essentially healed the scratch at 48 hours, while the SW group left a large unhealed area.

The above results indicate that SW can significantly inhibit the proliferation of HaCaT cells and the closure of scratches. After coculture with hADSCs, the proliferation and migration ability of the cells was significantly restored. Therefore, we believe that hADSCs promote the healing of seawater-soaked wounds by inducing the proliferation and migration of epidermal cells.

### 3.4. Ki67 and CK19 Expression Levels in Seawater-Immersed Wounds after hADSC Treatment

**Figure 5:**
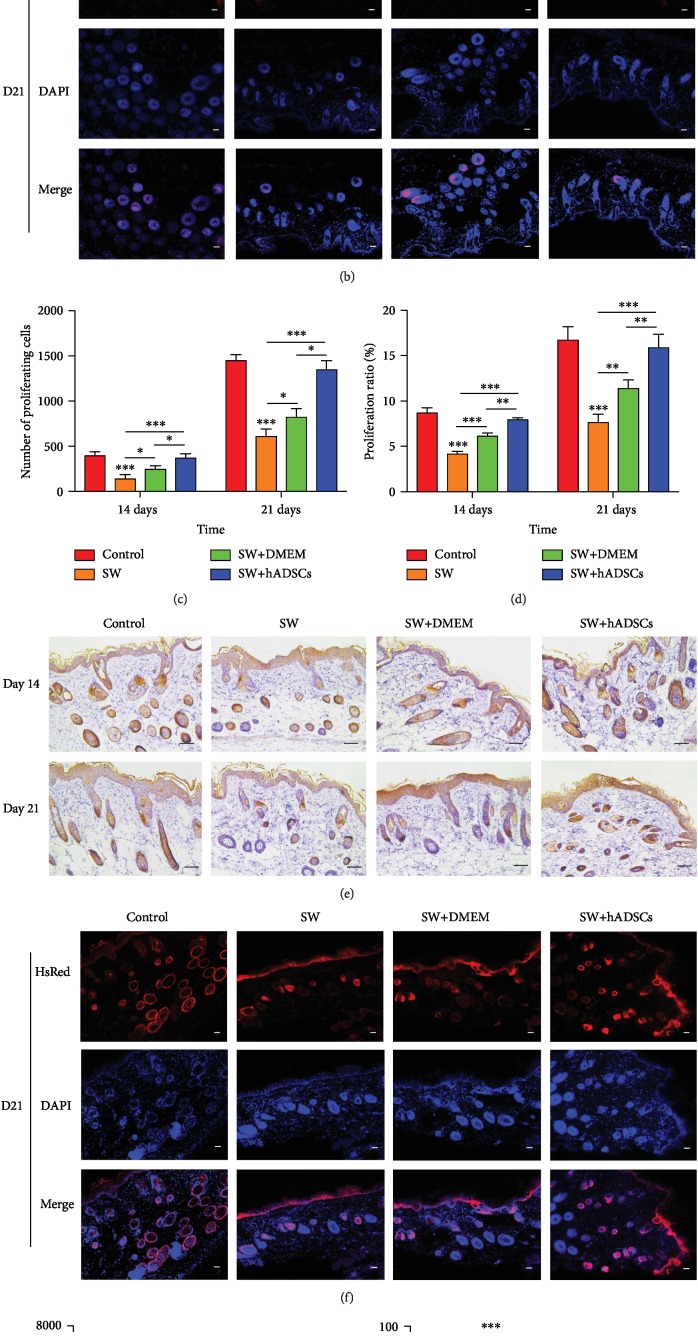
Ki67 and CK19 expression during wound repair after SW and hADSC treatments. The number and proportion of proliferating cells, as determined by Ki67 immunohistochemical and immunofluorescence (red) (a–d). The number and proportion of skin stem cells, as determined by cytokeratin 19 immunohistochemical and immunofluorescence (red) (e–h). DAPI staining shows the nuclei of tissues (blue). The number and proportion of proliferating cells or skin stem cells in the control group and the SW+hADSC group were significantly higher than those in the SW group. Scale bars indicate 100 *μ*m. ^∗^*P* < 0.05, ^∗∗^*P* < 0.01, and ^∗∗∗^*P* < 0.001.

Previous studies have shown that Ki67 is an important nuclear protein for cell proliferation, and CK19 is an important membrane protein for skin stem cell proliferation [16, 20, 23]. The proliferation of cells (especially skin stem cells) plays an important role in the repair of skin wounds. Therefore, we further analyzed the regulatory effects of SW and hADSCs on Ki67 and CK19 in wound healing cells. Immunohistochemical and immunofluorescence analyses of skin sections in the mouse model were used to evaluate wound repair. First, immunofluorescence analysis of frozen sections (Figure 4(e)) showed that hADSCs differentiated into skin stem cells to promote wound healing. Second, immunohistochemical staining of Ki67 and red fluorescent labeling of proliferating cells were performed, and the number and proportion of proliferating cells were calculated. The results (Figures 5(a)–5(d)) showed that the number and proportion of proliferating cells in the control group and the SW+hADSC group were significantly higher than those in the SW group (*P* < 0.001). CK19 immunohistochemistry and immunofluorescent labeling of skin stem cells (Figures 5(e)–5(h)) showed that the number and proportion of skin stem cells in the control group and the SW+hADSC group were significantly higher than those in the SW group (*P* < 0.001).

The above results indicate that SW immersion significantly reduces the expression of Ki67 and CK19 in skin wound repair and increases the damage of skin wounds, while hADSCs promote the expression of Ki67 and CK19 in skin cells, reduce the effect of SW on wounds, and accelerate wound healing.

### 3.5. hADSCs Affect MEK/ERK Signaling Pathway Activation in SW-Treated Wounds

**Figure 6:**
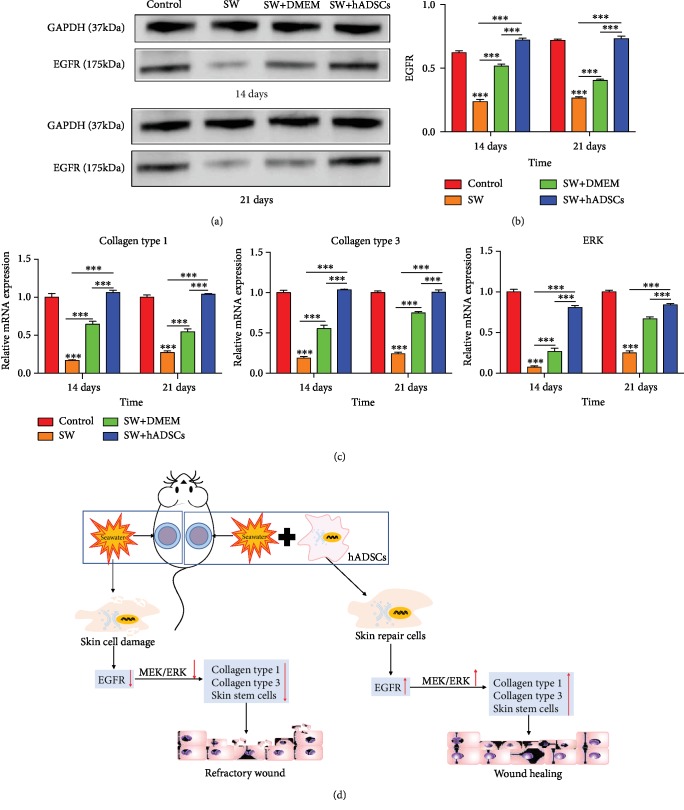
hADSCs affect MEK/ERK signaling pathway activation in SW-treated wounds. The expression levels of EGF protein (a, b) and collagen type 1, collagen type 3, and ERK mRNA levels (c) in the control group and the SW+hADSC group were significantly higher than those in the SW group and the SW+DMEM group. A schematic diagram shows how SW and hADSCs regulate wound healing through the MEK/ERK signaling pathway (d). ^∗^*P* < 0.05, ^∗∗^*P* < 0.01, and ^∗∗∗^*P* < 0.001.

We further analyzed the potential mechanism behind hADSC functions in SW-treated wounds. Previous literature has shown that wound repair is closely related to the EGF pathway [24], so we hypothesized that hADSCs induce proliferation and migration of wound repair cells by activating EGFR. The expression levels of EGF protein (Figures 6(a) and 6(b)) indicate that hADSCs promote the expression of EGFR in skin wounds. Further, previous studies have shown that EGFR can regulate the activity of the MEK/ERK pathway [25]. Therefore, we examined the activity of the MEK/ERK pathway in repairing wounds. The results show that collagen type 1, collagen type 3, and ERK mRNA (Figure 6(c)) levels in the control group and the SW+s group were significantly higher than those in the SW group and the SW+DMEM group (*P* < 0.001). These results suggest that hADSCs can activate the MEK/ERK pathway by regulating EGFR.

## 4. Discussion

The marine industry has been developing in recent years, and marine workers are prone to various open injuries [26]. Several previous studies have reported that SW immersion causes large amounts of inflammatory factors, such as IL-8, TNF, and NO, to be released from the injured area, causing multiple organ damage in the body and increasing the probability of disseminated intravascular coagulation, which ultimately leads to an increase in mortality [4, 27–31]. However, the effects of SW immersion on wound repair of full-thickness skin defects and its mechanism have not been reported in the literature. In our experiments, we established an SW immersion *in vivo* model and observed that the healing efficiency of SW-soaked wounds is significantly delayed. We also found that the proliferation and migration ability of epidermal cells was significantly inhibited after artificial SW treatment using *in vitro* experiments. The above results indicate that SW immersion can significantly delay wound healing.

ADSCs can inhibit the inflammatory response of wounds and promote the vascularization and epithelialization of wounds by releasing a variety of growth factors, such as EGF, HGF, and TGF-*α*, and factors that inhibit proinflammatory and enhance anti-inflammatory effects, such as IL-10 [32, 33]. Therefore, we hypothesize that ADSCs can significantly promote the repair of SW-soaked wounds. hADSCs were injected into the base of the SW-soaked wound model, and the results showed that hADSCs could reduce the damage of skin wounds caused by SW and accelerate the healing of SW-immersed skin wounds. Further, we cocultured epidermal cells with hADSCs in an artificial SW treatment model. The results confirmed that hADSCs could significantly promote the proliferation and migration of SW-treated epidermal cells. In addition, previous studies have reported that ADSCs can differentiate into epithelial stem cells to promote skin wound repair, which was shown by using specific histochemical markers (CK19) of repaired tissues that could distinguish the source of the healing cells [13]. Our research also confirmed this phenomenon.

As an important cell type in tissue regeneration engineering, ADSCs have shown remarkable repair effects in promoting skin wound healing (especially chronic skin wound healing) through various mechanisms, such as multidirectional differentiation and paracrine pathway activation [5, 34–36]. Moreover, EGF secreted by ADSCs is the most important growth factor for skin reepithelialization. Previous studies have shown that the expression of EGF can activate the MEK/ERK signaling pathway and promote cell proliferation and migration [25]. We found that there was a prolonged block of EGF expression in skin wounds following SW immersion, which further hindered the activation of the MEK/ERK signaling pathway and delayed the reepithelialization of skin wounds, as shown in Figure 6(d). However, ADSCs can induce the expression of EGF in wounds and activate the MEK/ERK signaling pathway to promote skin stem cell proliferation and migration.

There were still some shortcomings in this study. The SW used in our experiment was aseptic artificial SW. We have not studied the effect of bacteria on wounds. However, if bacteria were added to SW, skin wounds were more likely to cause serious contamination, and skin wounds would be more likely to form chronic wounds. Chen et al. [37] compared the cytokine levels of stem cell exosomes and stem cells by antibody array technology. The results showed that stem cell exosomes were significantly enriched with EGF compared with the stem cells themselves. Therefore, we hypothesized that in coculture systems, ADSCs secrete exosomes rich in EGF that promote the proliferation and migration of epithelial cells. Furthermore, we plan to compare the repair effects of ADSC and ADSC exosomes on SW-immersed skin wounds to explore the role of stem cell exosomes in wound repair. Subsequently, single cell sequencing technology will be used to compare the cells and their molecular components in repaired tissue to more thoroughly explore repair mechanisms.

## 5. Conclusions

In conclusion, we found that ADSCs could differentiate into skin stem cells and promote the accumulation of autologous skin stem cells by activating the EGFR/MEK/ERK pathway, thus achieving the recovery of SW-immersed wounds. Our results provide a new treatment for the regeneration of seawater-immersed skin wounds.

## Figures and Tables

**Table 1:** Primers used for qRT-PCR.

Gene	Forward primer (5′-3′)	Reverse primer (5′-3′)
GAPDH	TGGCCTTCCGTGTTCCTAC	GAGTTGCTGTTGAAGTCGCA
Col1	GGTGAGCCTGGTCAAACGG	ACTGTGTCCTTTCACGCCTTT
Col3	CTGTAACATGGAAACTGGGGAAA	CCATAGCTGAACTGAAAACCACC
ERK	GGTTGTTCCCAAATGCTGACT	CAACTTCAATCCTCTTGTGAGGG

## Data Availability

We declare that the materials described in the manuscript, including all relevant raw data, will be freely available to any scientist for use in noncommercial applications, without breaching participant confidentiality.

## Abbreviations

CCK8: Cell counting kit-8
DMEM: Dulbecco's Modified Eagle's Medium
EdU: 5-Ethynyl-2′-deoxyuridine
EGF: Epidermal growth factor
FBS: Fetal bovine serum
hADSCs: Human adipose derived stem cells
HE: Hematoxylin and eosin
HGF: Hepatocyte growth factor
IL: Interleukin
OD: Optical density
PBS: Phosphate buffered saline
P/S: Penicillin/streptomycin solution
SW: Seawater
TGF-*α*: Transforming growth factor-*α*
VEGF: Vascular endothelial growth factor.
